# Editorial: Prediabetes - early interventions and prevention in insulin resistance

**DOI:** 10.3389/fnut.2024.1434569

**Published:** 2024-06-03

**Authors:** Ntethelelo Sibiya, Alina Kurylowicz, Andile Khathi

**Affiliations:** ^1^Pharmacology Division, Faculty of Pharmacy, Rhodes University, Grahamstown, South Africa; ^2^Mossakowski Medical Research Institute, Polish Academy of Sciences, Warsaw, Poland; ^3^School of Laboratory Medicine and Medical Sciences, College of Health Sciences, University of KwaZulu-Natal, Durban, South Africa

**Keywords:** prediabetes, early interventions, prevention, insulin resistance, diet

Prediabetes is regarded as a state of intermediate hyperglycaemia that exists between normoglycaemia and the onset of type 2 diabetes mellitus (T2DM). This condition is characterized by impaired fasting glucose (IFG), impaired glucose tolerance (IGT), and elevated glycated hemoglobin concentrations without reaching the threshold for a diagnosis of T2DM (1, 2). Due to the asymptomatic nature of the condition, prediabetes is generally regarded as under-diagnosed (1). Recent estimates show that the prevalence of prediabetes was at 280 million in 2013, it is expected to increase to 398 million by the year 2030 (3). The increase prevalence of this condition has been directly correlated to the increased consumption of high-calorie diets consisting of refined carbohydrates and saturated fats as well as sedentary lifestyles in modernized and urbanized areas (4). Other risk factors associated with prediabetes include age, obesity and other pathological conditions that can result into a state insulin resistance (4). Using a diet-induced animal model of prediabetes, recent studies have shown that the complications often observed in overt T2DM may begin during the prediabetic state (5–9). Using the same model, a study has demonstrated that prediabetes is a risk factor for developing preeclampsia (Ludidi et al.). In another recently published study using the same model, prediabetes was reported to result in higher fibrinolysis and endothelial dysfunction when compared to non-prediabetic animals (Gumede and Khathi). With several studies showing that prediabetes is reversible, this condition has since emerged as a potential avenue for dietary and pharmacological intervention to prevent the onset of T2DM (10).

The management of overt T2DM and the associated complications places a huge financial burden on health care systems globally as various pharmacological agents that range from, but not limited to, anti-hyperglycaemics, cholesterol lowering agents, anti-coagulants and antihypertensives are often required. Therefore, the prevention and management of prediabetes perhaps through dietary intervention and natural products could be paramount in alleviating the costs associated with overt T2DM. A recently completed clinical trial (NCT03222791) demonstrated a significant change in metabolic profile and gut microbiota composition after a six-month dietary intervention through a personalized postprandial glucose-targeting diet or the standard of care Mediterranean diet (11). This trial further highlighted the impact of dietary intervention on the improvement of microbiota, perhaps influencing better metabolic outcomes. Without trivializing the effectiveness of dietary intervention, however it requires strict adherence and high motivation, hence probiotic as an alternative therapeutic intervention could serve a similar purpose interms of improving gut microbiota (11). For further understanding, a randomized clinical trial protocol aiming to determine the effectiveness of healthy diet in early pregnancy stages in preventing gestational diabetes mellitus has also been registered (Bernier et al.). This Research Topic briefly consolidates some cutting-edge research on recent strides focused on the prevention and management of prediabetes through dietary interventions and natural products.

A conflicting evidence has been presented on the effect of dietary fats including monosaturated (MUFA) and polysaturated fats (PUFA), thus necessitating larger scale populations studies. A recently published study by Jiang et al. based on a nationally representative population sample of the United States found that dietary MUFA and PUFA (18:2 and 18:3) intake to be associated with a decreased incidence of prediabetes and T2DM especially in young adults. The plant-based diet has also gained momentum in the prevention and managing prediabetes. Although there is evidence of plant-based diet benefits, however there are some inconsistencies. For these reasons more epidemiological looking at the association between active ingredient, i.e., flavonoids from plant-based diets and the risk of prediabetes are of paramount importance. A recently published study from a completed large scale survey suggests that higher intake of flavonoids is associated with a decrease in the risk of developing prediabetes which also correlates with previous data that showed beneficial effects of flavonoids in overt T2DM (Zhou et al.). Flavonoids are the category of bioactive compounds, abundantly present in several plant-originated foods, including fruits, vegetables, herbs, and any other edible plant parts. In quest of further understanding the beneficial role of natural compounds of plant-origin, Chen et al. aimed to consolidate evidence of various natural compounds effect on prediabetes and associated mechanisms of action. In a recently published review paper, natural compound classes such as phenolic compounds, flavonoids, terpenoids, alkaloids, glycosides, quinones, lactones, and saponins, natural compounds showed varying biological and pharmacological activities for prediabetes (Chen et al.). The evidence above, illuminates the beneficial effect of dietary intervention and natural compounds on the prevention and management of prediabetes. To expedite and validate our understanding on the effectiveness of diet-based interventions, clinical trials are however required. Another diet-based intervention which is gaining momentum in glycaemic control is intermittent fasting as it has been shown to show therapeutic effects. Recent studies have reported on several intermittent fasting strategies that include twice-per-week fasting, fasting-mimicking diets, time-restricted eating, and periodic fasting (12). A meta-analysis conducted by Xiaoyu et al. suggests that the twice per week fasting strategy affords better glycaemic control when compared with other strategies in the same category. Literature evidence demonstrates intermittent energy restriction to be efficacious in preventing and managing both prediabetes and T2DM while further showing protective benefits that extend beyond glycaemic control (13). Interestingly, a recent meta-analysis suggests that intermittent energy restriction is slightly more effective than the continuous counterpart, however, long term studies are still necessary (14). Considering that this is a relatively emerging field of study, more clinical studies and refinements are required to shape guidelines and policy development toward implementing this strategy in the prevention and management of prediabetes. Taken together, we envisage that the collection of works in this Research Topic will hopefully inspire and accelerate preventative and therapeutic strategies toward the management and possible treatment of prediabetes.

## Author contributions

NS: Conceptualization, Validation, Writing – original draft, Writing – review & editing. AKu: Conceptualization, Validation, Writing – review & editing. AKh: Conceptualization, Validation, Writing – original draft, Writing – review & editing.
